# The Efficiency of Deoxynivalenol Degradation by Essential Oils under In Vitro Conditions

**DOI:** 10.3390/foods8090403

**Published:** 2019-09-11

**Authors:** Adam Perczak, Krzysztof Juś, Daniela Gwiazdowska, Katarzyna Marchwińska, Agnieszka Waśkiewicz

**Affiliations:** 1 Department of Chemistry, Poznań University of Life Sciences, Wojska Polskiego Street 75, 60-625 Poznań, Poland; agat@up.poznan.pl; 2 Department of Natural Science and Quality Assurance, Faculty of Commodity Science, Poznań University of Economics and Business Poznań, Niepodległości Avenue 10, 61-875 Poznań, Poland; krzysztof.jus@ue.poznan.pl (K.J.); katarzyna.marchwinska@ue.poznan.pl (K.M.)

**Keywords:** deoxynivalenol, essential oils, reduction, mycotoxin, food safety, LC-MS/MS

## Abstract

Essential oils (EOs) are complex natural products of plant origin and exhibit different desirable, e.g., antimicrobial properties. Their growth inhibition effect on the pathogenic fungi of the genus, *Fusarium*, which forms deoxynivalenol (DON), has been documented. DON is the most common contaminant of grains and their products, causing strong emetic effects after their consumption. The aim of the study was to investigate the ability of selected EOs to degrade DON under in vitro conditions, using various incubation terms. The impact of a different temperature, pH, incubation time, mycotoxin, and essential oil concentration was tested. The results indicate that the kind of EO influences the effectiveness of mycotoxin level reduction, and the most effective EOs were palmarosa and lemon oils. A higher reduction of DON content by EOs was achieved after 24 h of the experiment (up to 72%), at a pH range between 3 and 6 and a temperature of 20 °C. Moreover, the effect of various doses of white and pink grapefruit and palmarosa EOs (100 and 200 μL/mL) on toxin level reduction was observed. The experiment confirmed that the selected EOs may be effective in DON reduction, as previously documented in experiments with zearalenone.

## 1. Introduction

Global development of the food industry affects the level of safety in the food chain [1]. The most important threats to food safety are microorganisms, including filamentous fungi and their toxic metabolites—mycotoxins. The presence of mycotoxins in food causes numerous health problems in humans and is connected with a number of negative effects on the economy, such as crop losses and a decrease in food quality [2]. Considering the above-mentioned information, it is obvious that the most important challenge in the food industry is to provide a sufficient quality of food and feed production, without negative effects on the environment, which is consistent with the concept of sustainable development [3]. However, current methods of mycotoxin decontamination are not sufficiently effective to ensure optimal safe standards in food and the feed chain [4]. Therefore, it would be appropriate to approach the problem related to mycotoxins in a complex and multi-stage manner, applying broadly defined integrated actions using different methods [5]. Biological decontamination methods, involving the use of microorganisms or natural, ecologically friendly substances, are especially interesting in this connection. 

The data from the literature indicate that microorganisms may degrade mycotoxins by enzymes or bind them through adsorption to the cell surface, which seems to be a very promising method. Different microorganisms were used in the process of mycotoxin, including deoxynivalenol, decontamination. For example, Garda-Buffon and Badiale-Furlong [6] applied *Aspergillus oryzae* and *Rhizopus oryzae* to eliminate DON and observed a decrease in mycotoxin content at a level of 74% due to the activity of *A. oryzea*. The authors suggest that the reduction of the mycotoxin concentration process is due to the fact that it is a source of carbon for the fermentation process. A number of studies have also been carried out to investigate the use of probiotic bacteria (lactic acid bacteria—LAB) in the adsorption of mycotoxins to the cell wall. Zou et al. [7] used several different strains of LAB to remove DON, and the most effective species was *Lactobacillus plantarum*. The mechanism of decontamination is probably connected with the adsorption of mycotoxins to the cell wall components, such as peptidoglycans and polysaccharides [8]. Moreover, some abiotic factors, such as a high temperature, may increase the durability of bonds between mycotoxins and bacterial cells.

Natural substances, such as essential oils, known for their antimicrobial activity, including antifungal properties, seem to be especially promising in the process of mycotoxin elimination. Essential oils (EOs) are highly concentrated aromatic volatile substances of plant origin, which include, in their composition, many different chemical groups, such as alcohols, ketones, aldehydes, and characteristic compounds, e.g., terpenes. A rich source of these substances, both in terms of quality and quantity, are angiosperms, such as *Asterales*, *Laurales*, *Magnoliales*, and *Zingiberales* [9,10]. The amount and the composition of EOs depends on several factors, including the species and part of the plant organs (including buds, flowers, leaves, stems, twigs, seeds, fruits, roots, wood, or barks), their quality, geographic region, cultivation methods, and extraction method [11,12]. The yield of essential oils from plants varies widely, and the broad range is 0.05–18.0% [13]. EOs are used in many industries, such as cosmetics, pharmaceuticals, perfumery, food, and agriculture. Of the more than 400 used essential oils, about 100 of them are used in aromatherapy [14,15].

Nowadays, EOs are under extensive evaluations as potential plant protection agents that could be used against pathogenic fungi in agriculture [16]. *Fusarium* filamentous fungi are considered to be some of the most important phytopathogenic species and are found in various agricultural regions of the world [17]. *Fusarium* spp. contribute to significant economic losses due to plant diseases and the biosynthesis of mycotoxins, which are secondary fungal metabolites and may cause many chronic and acute, e.g., nephrotoxic, carcinogenic, teratogenic, genotoxic, or immunosuppressive, effects on humans and animals [18,19,20]. According to estimated data from FAO/WHO, at least 25% of the world’s crops are contaminated each year by mycotoxins, which causes significant annual losses in food and feed product [21]. Some data suggest that, on a global level, about 30% or more of feed samples are co-contaminated [22]. 

Among the many toxic secondary metabolites of *Fusarium*, deoxynivalenol (DON) is one of the most important mycotoxins (along with zearalenone and fumonisins), from the point of view of food safety, and is produced mainly by *Fusarium graminearum* and *F. culmorum* [23,24]. Factors promoting the formation of this mycotoxin include a specific temperature range (21–25 °C) and humidity, depending on the *Fusarium* species [25]. DON is also called a vomitoxin due to the response occurring in humans and animals, following the consumption of food or feed containing this metabolite. The primary sources of DON are cereals, including wheat, barley, maize, and oat, as well as buckwheat, sorghum, and processed plant-origin food, such as flour, bread, pasta, beer, and malt [26,27,28]. DON and its derivatives may pass to the human organism through the consumption of contaminated food or water, through inhalation or through the skin [29,30]. The toxicity of DON is associated with the presence of the 12,13-epoxy ring, unsaturated bond at C9–C10 and hydroxyl group in appropriate positions in its structure [31]. The epoxide group is considered as an essential factor determining DON toxicity [32] since the literature data indicate that DON may be transformed in animals into 12,13-de-epoxy-DON (DOM-1), a metabolite with lower toxicity than DON [33].

Due to the harmful effect on animal and human health, as well as to ensure food and feed safety, there is a constant need to control fungal growth and mycotoxins production in crops. Chemical fungicides are often used to control *Fusarium*. However, despite their effectiveness and ease of application, they have not gained sufficient recognition. Phytotoxigenic fungi have become resistant to fungicides. Moreover, they reduce beneficial organisms and pose a health hazard to consumers. This has led to a search for alternative methods of controlling *Fusarium* and its metabolites, with the application of effective biodegradable fungicides and products containing active substances of natural origin or antagonistic microorganisms [34,35,36]. Agrochemicals, based on essential oils, are easily degraded, and they are therefore considered to be environmentally friendly. EOs may also be qualified as “GRAS” (generally recognized as safe) and are used as food additives and preservatives [37,38]. The development of alternative plant protection methods, based on chemicals of natural origin, can reduce the level of mycotoxins in food. So far, in the available literature, there are few reports on the ability of selected essential oils for zearalenone, fumonisin B_1_, or ochratoxin A elimination [39,40,41].

Considering the above-mentioned information, the aim of our research was to evaluate the effect of different parameters (pH, temperature, incubation time, and the concentration of essential oil and a mycotoxin) on the reduction of deoxynivalenol content by selected essential oils.

## 2. Materials and Methods 

### 2.1. Chemicals

The DON standard, LC/MS-grade organic solvents and other reagents were purchased from Sigma-Aldrich (Steinheim, Germany). The distillated water used for the studies was purified using a Milli-Q system (Millipore, Bedford, MA, USA). A DON stock solution was prepared in methanol, at a concentration of 1 mg/mL, and was stored at −20 °C. Three buffer solutions, based on citrate (pH 3 and 6) and ammonia (pH 9), were used in the experiment.

### 2.2. Essential Oils

Seven EOs used in the laboratory work studies were reviewed: cinnamon from bark (*Cinnamomum zeylanicum*, Indonesia, composition: cinnamic aldehyde ≤ 70%, eugenol ≤ 4.4%, linalool ≤ 2.6%, limonene ≤ 1.1%, benzyl benzoate ≤ 1.1%, benzaldehyde 0.5%, cinnamic alcohol ≤ 0.4%, cuminaldehyde ≤ 0.2%), cinnamon from leaf (*Cinnamomum zeylanicum*, Sri Lanka, composition: eugenol ≤ 83%, benzyl benzoate ≤ 4%, linalool ≤ 3.1%, cinnamal ≤ 1.8%, limonene ≤ 0.5%, cinnamyl alcohol ≤ 0.2%, benzaldehyde ≤ 0.2%, coumarin, carvone and geraniol ≤ 0.1%), lemon peel (*Citrus limonum*, Italy, composition: limonene 72%, citral ≤ 3.5%, linalool ≤ 0.5%, furocoumarins ≤ 0.45%, geraniol ≤ 0.1%, citronellol ≤ 0.01%), white grapefruit peel (*Citrus grandis*, Argentina, composition: limonene 96%, linalool 0.15%, citral 0.5%), pink grapefruit peel (*Citrus paradisi*, Argentina, composition: limonene 96.5%, linalool 0.03%, citral 0.5%), eucalyptus leaf (*Eucalyptus radiata*, China, composition: limonene about 70%, limonene 5–10%, G-terpinene ≤ 5%, α-pinene ≤ 5%, β-pinene ≤ 5%), and palmarosa leaf (*Cymbopogon martinii*, India, composition: geraniol 85%, linalool 2–3%, limonene 1%, citral 1%). Concentrated EOs were obtained from Ecospa Rita Kozak-Chaber Artur Chaber s.c., Poland and from Zrób Sobie Krem, Kosmetyki Naturalne Katarzyna Damętka, Zomerfeld, Poland. EO solutions were prepared by mixing the commercial product with water and Tween 80 (as an emulsifying agent). Different concentrations of EOs were used (100 and 200 μL/mL), depending on the experiment being conducted.

### 2.3. Preparation of Samples

The impact of the selected EOs was examined by mixing the DON solution with an EO solution to achieve the final toxin concentration of 1 μg/mL. A single experimental sample contained 100 μL of EO, 100 μL of Tween 80, and 10 μL of the DON solution and was filled up with a buffer solution (pH 6) to a volume of 1 mL. The proportions of the main components (EOs and DON) and the type of buffer (pH 3, 6 or 9) were different, depending on the experiment being conducted. Samples were shaken and stored at 20 °C, and LC-MS/MS analysis was performed after 72 h.

### 2.4. Effect of the Incubation Time, Temperature, pH, Concentration of Eos, and/or DON on the Mycotoxin Level Reduction

In order to establish the effect of different factors on DON content reduction by EOs, five experiments were conducted. The reduction of the deoxynivalenol content by EOs at 0, 24, and 72 h of incubation was investigated. Different process conditions, including the concentration of essential oils (100 and 200 μL/mL), temperature of the sample incubation (4 and 20 °C), along with the pH (3, 6, and 9) and concentration of the toxin (0.5, 1, and 5 μL/mL of DON), were tested after 72 h of incubation. 

### 2.5. DON Analysis by the LC-MS/MS Method

After incubation, 1 mL of each mixture was homogenized for 2 min with acetonitrile: water (80:20, *v*/*v*). Deoxynivalenol was extracted and purified by a DON Test WB HPLC column (Vicam, Milford, CT, USA), according the manufacturer’s procedure. The elute was evaporated to dryness at 40 °C under a stream of nitrogen, and it was then dissolved in 500 µL of acetonitrile and filtered through a syringe filter of 0.2 µm mesh, before chromatographic analysis.

The analytical system consisted of the Aquity UPLC chromatograph (Waters, Manchester, MA, USA), coupled with an electrospray ionization triple quadrupole mass spectrometer (TQD) (Waters, Manchester, MA, USA). A Waters ACQUITY UPLC HSS T3 (100 × 2.1 mm/ID, with a particle size of 1.8 µm) (Waters, Manchester, MA, USA) was used for chromatographic separation, with a flow rate of 0.35 mL/min at room temperature. A gradient elution was applied using water buffered with 10 mM ammonium acetate (A) and acetonitrile (B). The solvent gradient was modified as follows: 0–2 min at 5% B, 2–7 min 55% B, and 9–15 min 90% B, with isocratic elution for 2 min, followed by a return to the initial conditions. The purity of the nitrogen used was >99%. The collision-induced decomposition was performed, using argon as the collision gas, with a collision energy of 14–22 eV. DON was quantitatively analyzed using multiple reaction monitoring and identified by comparing the retention times and m/z values, obtained by MS and MS2, with the mass spectra (297.3/249.1) of the corresponding standard, tested under the same conditions. All samples were analyzed in triplicate.

### 2.6. Statistical Analysis

The data were presented as the mean (±standard deviation) from three replicates, and the results were subjected to Tukey’s test at *p* < 0.05 to test for significant differences between different tested samples. The effect of using various conditions (temperature, pH, and the concentration of EOs and toxins) on the reduction of DON concentration was examined by multivariate analysis of variance (ANOVA). The statistical analysis was carried out using STATISTICA for Windows, version 10.

## 3. Results

The effect of selected essential oils on deoxynivalenol reduction under differentiated in vitro conditions, including the influence a mycotoxin and essential oil concentrations, as well as environmental conditions, such as the incubation time, temperature, and pH, was investigated. The selection of EOs for experiments was based on the results of previously published studies [41]. The degree of deoxynivalenol reduction was determined by the LC-MS/MS method. 

### 3.1. The Effect of DON Concentration on Its Level Reduction by EOs 

**Figure 1:**
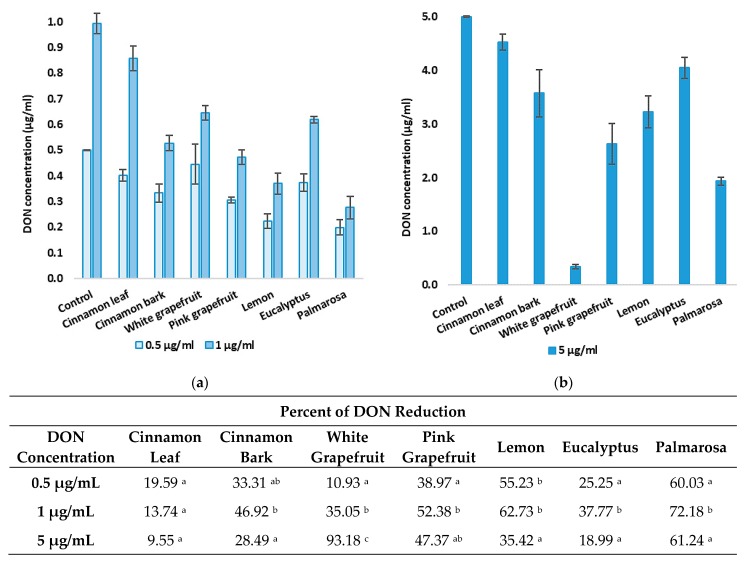
The effect of deoxynivalenol concentration ((**a**)—0.5 and 1 µg/mL; (**b**)—5 µg/mL) on its content reduction by EOs. The experiment was conducted up to 72 h. Data were analyzed by Tukey’s test at *p* < 0.05 (^a^, ^b^, ^c^—significantly different in column).

The first stage of experiments considered the effect of essential oils on the concentration decrease of deoxynivalenol, depending on the various concentrations of a mycotoxin (Figure 1). Three different concentrations of DON—0.5, 1.0, and 5 μg/mL—have been taken into consideration. The highest percentage reduction of DON concentrations was observed at the initial concentration of 1.0 μg/mL. The reduction degree reached 72.28% for palmarosa, 62.73% for lemon, 54.49% for eucalyptus, and 52.38% in the case of pink grapefruit EO. The only exception was white grapefruit EO, which reduced the DON content by up to 93.18% at the initial concentration of 5.0 μg/mL, while a significantly lower efficacy was observed in samples at the initial concentrations of 0.5 and 1.0 μg/mL. It is worth noting that there was no observed significant difference in the efficiency of DON level reduction by cinnamon leaf EO, depending on the toxin concentration. Considering the obtained results, the concentration of 1.0 μg/mL was used in the following experiments. 

### 3.2. Effect of Different Incubation Time on DON Level Reduction by EOs

**Figure 2:**
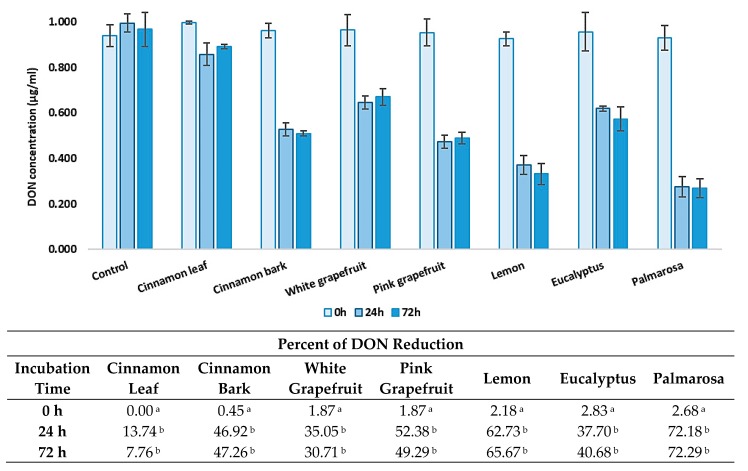
Reduction of DON concentration by essential oils at different incubation time. The experiment was conducted up to 72 h at 20 °C. Data were analyzed by Tukey’s test at *p* < 0.05 (^a^, ^b^—significantly different in column).

The next part of the study consisted of the use of examined EOs to test their effect on DON reduction, depending on the incubation time. The toxin concentration was determined immediately after mixing the EOs and DON, as well as after 24 h and 72 h of incubation (Figure 2). All tested oils reduced the concentration of the toxin. Immediately after preparing the samples of the toxin with the EOs, the degree of DON reduction did not exceed 3%. However, after 24 h and 72 h of incubation, a significant decrease of the mycotoxin concentration was observed. Palmarosa and lemon essential oils were the most effective. The amount of the toxin was reduced by up to 72.18% after 24 h and 72.29% after 72 h by palmarosa oil and by up to 62.73 after 24 h and 65.67% after 72 h by lemon oil. Additionally, pink grapefruit, eucalyptus, and cinnamon bark efficiently reduced the concentration of DON. It is worth noting that the high degree of DON reduction was observed after 24 h, while after 72 h, the degree of DON reduction remained at a similar level.

### 3.3. Effect of EO Concentration on DON Level Reduction

**Figure 3:**
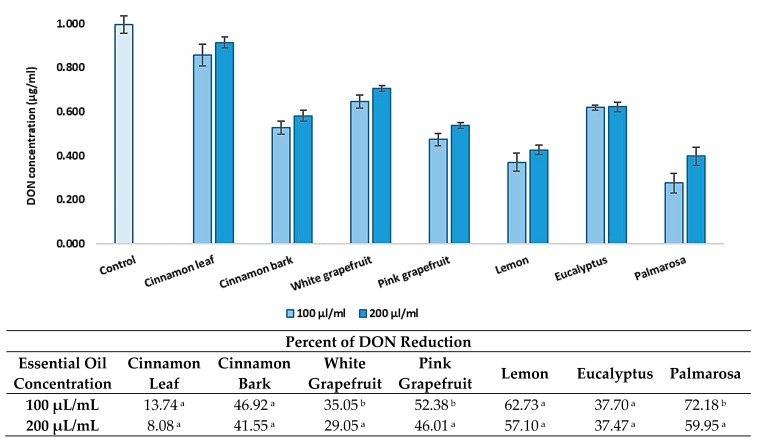
The effect of the essential oils concentration on reduction of deoxynivalenol concentration. The experiment was conducted up to 72 h at 20 °C. Data were analyzed by Tukey’s test at *p* < 0.05 (^a^, ^b^—significantly different in column).

In the subsequent stage, two different doses (100 and 200 μL/mL) of each tested EO (Section 2.2) were used to investigate the effect on the concentration decrease of DON (Figure 3). The conducted experiment confirmed the high efficiency of palmarosa, lemon, and pink grapefruit essential oils in the reduction of the DON concentration. For four of the seven EOs, the dose of the oil did not influence the toxin level reduction degree. However, in the case of palmarosa and white and pink grapefruit EOs, a significant decrease in DON reduction effectiveness was noted.

### 3.4. Effect of pH and Temperature on DON Level Reduction by EOs

**Figure 4:**
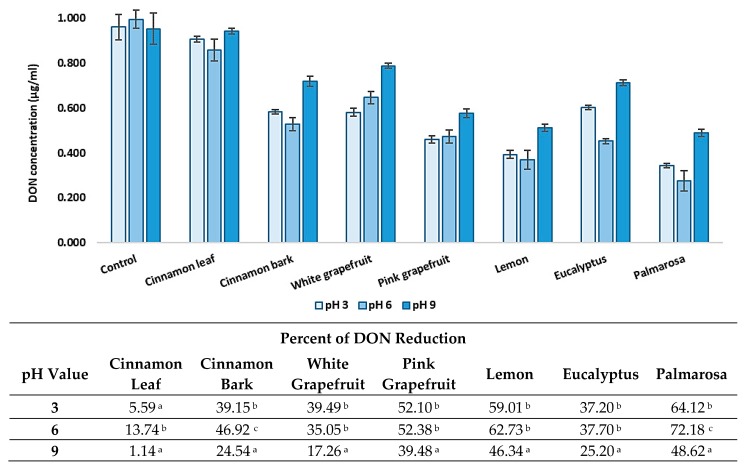
The effect of pH on reduction of deoxynivalenol concentration at 20 °C. The experiment was conducted up to 72 h. Data were analyzed by Tukey’s test at *p* < 0.05 (^a^, ^b^, ^c^—significantly different in column).

**Figure 5:**
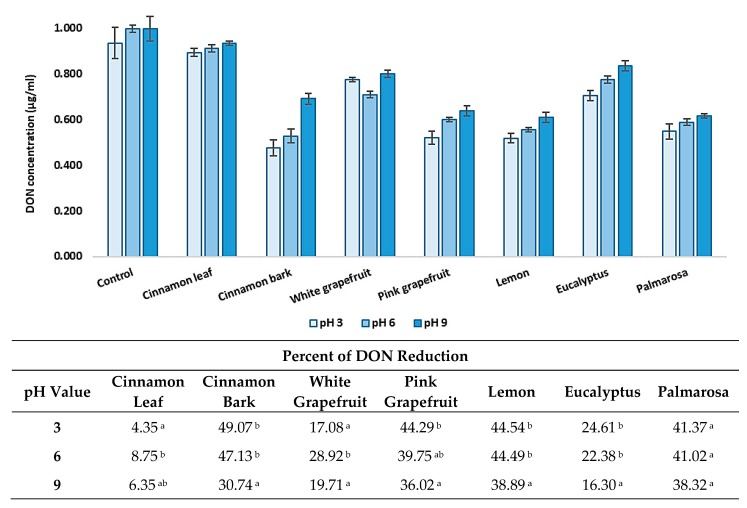
The effect of pH on reduction of deoxynivalenol concentration at 4 °C. The experiment was conducted up to 72 h. Data were analyzed by Tukey’s test at *p* < 0.05 (^a^, ^b^, ^c^—significantly different in column).

The content of deoxynivalenol decreased under various environmental conditions, including pH values of 3, 6, and 9 and incubation temperatures 20 °C and 4 °C, were carried out (Figure 4 and Figure 5). All tested parameters influenced the effectiveness of toxin reduction. However, the results depended on the type of EO and the testing conditions. Considering the reduction degree of DON by the tested EOs, depending on the pH value, it could be stated that, generally, the highest reduction of the toxin amount was observed at pH 3 and 6, while at pH 9, the reduction degree was usually significantly lower. At the temperature of 20 °C, palmarosa and cinnamon bark EOs reduced the toxin amount with the highest efficacy at pH 6, while a significantly lower reduction degree was observed at pH 3. The lowest effect was observed at pH 9. White and pink grapefruit, as well as lemon and eucalyptus EOs, reduced the DON concentration to a similar degree at pH 3 and 6, with a significantly lower efficiency at pH 9. In turn, the reduction degree of the DON amount by eucalyptus was similar in the range of pH 3–9, while the cinnamon leaf EO was the most effective at pH 6 and demonstrated a significantly lower and similar efficacy at pH 3 and 9. At the temperature of 4 °C, the results were also differentiated. Cinnamon bark, pink grapefruit, lemon, and eucalyptus essential oils were the most effective in DON content decreased at pH 3 and 6, with a significantly lower efficiency at pH 9. Cinnamon leaf and white grapefruit EOs caused the highest reduction of the DON amount at pH 6, while at pH 3 and 9, the elimination degree was significantly lower or similar. In turn, pink grapefruit was the most effective at pH 3, with a lower efficiency at pH 6 and 9, while the palmarosa EO reduced the DON content to a similar degree in the range of pH 3–9. Considering the effect of temperature and pH on the level reduction of DON content, it is worth underlining that the highest percentage reduction was observed at 20 °C and in the range of pH 3–6.

## 4. Discussion

*Fusarium* species produce a range of mycotoxins, among which deoxynivalenol is the most often detected. It is commonly found in wheat, barley, corn, and other cereal crops and food products. A serious problem connected with the presence of DON and other mycotoxins in food chain is their high stability, which means that the compounds are not degraded during technological processes such as pasteurization and sterilization and may contaminate the food and feed. Therefore, effective methods of fungal toxins elimination at different stages of food and feed production are searched. 

Essential oils have been attracting more interest in recent years, as many of them are recognized as natural, biodegradable, and non-toxic compounds, so they can be taken into consideration as an eco-friendly and safe alternative to chemical fungicides or preservatives. As literature data indicate, EOs may be used to inhibit the growth of fungi and prevent mycotoxin production during plant growth in the field, during storage and during food or feed production. 

Antifungal and antimycotoxigenic activity of EOs in relation to different fungi including *Fusarium* genus has been described by many authors. Elhouiti et al. [42] used the EO from *Rhanterium adpressum* to inhibit the growth of *F. graminearum* and *F. culomorum*. In turn, Amini et al. [43] showed that EOs obtained from *Zataria multiflora*, *Thymus vulgaris*, and *Thymus kotschyanus* inhibited *F. graminearum.* Other studies showed that, regardless of the concentration of geranium and rosewood oil (in the range 0.125–2.0%), complete inhibition of the growth of the two tested *F. graminearum* isolates was noted, whereas the action of the other two oils (lemon and rosemary) depended on their concentration and isolate of *Fusarium graminearum* [44]. In addition to testing the activity of the above EOs against *F. graminearum*, research was also carried out using thymol—a natural plant-derived compound, which also strongly inhibited conidial production and hyphal growth [45]. In studies with isolates of *Fusarium verticillioides*, antifungal and antimycotoxigenic properties showed essential oils from *Cuminum cyminum*, *Curcuma longa*, *Rosmarinus officinalis*, *Thymus vulgaris*, *Mentha arvensis*, and *Zingiber officinale* [16,46,47,48,49]. The inhibition of fungal biomass and fumonisins biosynthesis was dependent on the concentration of tested EOs. In addition, the antifungal action of essential oils was also tested on the less important species of *Fusarium*: *F. solani* [50], *F. equiseti* [51], and *F. oxysporum* [52,53]. The inhibition of fungal growth is often related to a decrease in mycotoxin production, which is highly desirable and helps to reduce the risk connected with the presence of secondary fungal metabolites in food or feed. Research conducted by Perczak et al. [54] demonstrated the high antifungal activity of *Cinnamonum zeylanicum* bark, *Cymbopogon martinii* leaves, and *Origanum vulgare* herb EOs toward *F. culmorum* and *F. graminearum*. Moreover, these EOs efficiently reduced the production of fungal secondary metabolites as zearalenone and trichothecenes. 

The presented work was concentrated on the problem of deoxynivalenol elimination by selected essential oils. It is worth noting that, so far, the data reporting the use of various EOs in reduction of mycotoxins are strongly limited, and there is a lack of similar data concerning deoxynivalenol. Seven EOs selected on the basis on earlier study [41] were investigated for the possibility of the reduction of DON content in the in vitro tests taking into account different conditions. All examined EOs decreased the concentration of DON, but the reduction degree was dependent on the EO kind and incubation conditions. Similar observations were reported for fumonisin B_1_ and zearalenone. Xing et al. [29] used different EOs in the reduction of fumonisin B_1_ (FB_1_) level and tested them under various conditions. The authors stated that the effectiveness of fumonisin B1 reduction depended on the EO kind and some examined factors, such as temperature, incubation time, and the dose of essential oil. The decrease rate of fumonisin B_1_, as reported by Xing et al. [40], increased with the increasing temperature (in the range of 20–35 °C) and with the increasing incubation time (up to 120 h). In turn, Perczak et al. [41] investigated the reduction of zearalenone content by various EOs. The decrease rate of zearalenone concentration also increased with the increasing incubation time (up to 72 h) as well as at a higher temperature. In the presented work, the reduction rate of deoxynivalenol content was also dependent on the used conditions. However, the results are not always consistent with those obtained for fumonisin B_1_ and zearalenone. Similar to works described above, we have observed a higher effectiveness of DON elimination at a higher temperature (20 °C), but considering the incubation time, a higher degree of DON elimination was usually observed after 24 h and did not increase after 72 h. Perczak et al. [41] also underline the effect of pH on reduction of zearalenone concentration, which was also observed in the presented work. Moreover, the dose of essential oil used in mycotoxin content reduction may influence the effectiveness of the process, as suggested by other authors [40,41]. The fumonisin B_1_ reduction rate by cinnamon EO was higher with the increasing content of the essential oil [40], while Perczak et al. [41] noticed that the dose of EOs effectively reduced zearalenone, depending on the type of EO. In the presented work, the dose of oil did not affect the level decrease of toxin in the case of four out of seven EOs. However, in the case of palmarosa, white and pink grapefruit EOs, better results were obtained with a lower dose of EO.

Comparing the available data, it could be stated that the reduction rate of individual mycotoxins is strongly dependent on the kind of essential oil. Fumonisin B_1_ was most effectively reduced by cinnamon bark, followed by citral, eugenol, eucalyptus, and camphor essential oils [40]. Zearalenone was degraded with the highest effectiveness by lemon, grapefruit, eucalyptus, and palmarosa oils [41]. In the presented work, the most effective EOs in DON level reduction were palmarosa, lemon, eucalyptus, and pink grapefruit EOs. Moreover, cinnamon bark effectively decreased DON concentration. The obtained results, as well as the data from the literature, prove that the reduction of mycotoxins is primarily dependent on the kind of EOs, which is probably connected with its chemical composition. 

The used commercial EOs differed in their composition. It is worth noting that EOs demonstrating the highest effectiveness in the reduction of DON content are rich in limonene, with the exception of palmarosa EO, where only a small amount is present. Lemon oil contains a high amount of monoterpene hydrocarbons (about 90%), with limonene (above 60%), *γ*-terpinene, and *β*-pinene being the first three major components. Pink grapefruit EO is characterized by a high amount of monoterpene hydrocarbons, of which limonene (above 90%), *β*-pinene, linalool, and *α*-terpinene are dominating. Moreover, pink grapefruit EO contains a lower amount of sesquiterpene hydrocarbons, aldehydes, alcohols, and esters than lemon oil [55,56]. Similarly, commercial eucalyptus essential oil is rich in limonene (about 70%), *α*-terpineol (about 10%), *α*-terpinyl acetate, and *α*-pinene [57]. In turn, the main component of palmarosa essential oil is geraniol (64–92%), followed by geranyl acetate, limonene, and epi-*α*-cadinol [58]. The white grapefruit EO, less efficient in DON elimination than the above-mentioned EOs, contains limonene as the main component (about 60%) followed by *β*-pinene and linalool [59,60]; however, the dominating compound is present in a lower amount than in the above-mentioned EOs. Taking into account that the composition of essential oils may play a role in the reduction of DON content, the interesting comparison can be made with cinnamon EO from bark and leaf, which strongly differ in their biological activity. The main component of cinnamon leaf essential oil is eugenol (about 90%), followed by bicyclogermacrene, α-phellanderene (1.9%), β-carryophyllene, aromadendrene, p-cymene, and 1,8-cineole. In turn, the main component of cinnamon bark EO is (E)-cinnamaldehyde (97.7%), δ-cadinene, α-copaene, and α-amorphene [61]. It is worth noticing that cinnamon bark EO efficiently reduced DON content, while cinnamon leaf EO demonstrated low activity. Although the mechanism of the reduction of DON content is difficult to explain, the composition of EOs seems to be crucial. For example, in the study of Singh et al. [61], (E-) cinnamaldehyde showed higher antimicrobial activity than eugenol, which may explain a higher biological activity of cinnamon bark EO in comparison with cinnamon leaf EO. It was also reported that some compounds found in EOs inhibit the growth of the fungi responsible for the formation of mycotoxins, such as DON, resulting in an impaired function of the cell membrane and mitochondria of various microorganisms [62,63]. It should be noted that there are no reports on the contribution of individual EO components to the decrease in deoxynivalenol concentration. However, many authors underline that biological action of essential oils is the result of the action of different components and their synergistic or antagonistic activity. 

The mechanism of the reduction of deoxynivalenol concentration is not yet explained. Because selective immunoaffinity columns were used in DON extraction and purification, possible derivatives or reaction products were not analyzed. Therefore, it is uncertain whether a degradation process took place. 

Finally, we consider the use of EOs in the different stages of the food production chain. Many formulations containing EOs are on the GRAS (Generally Recognized As Safe) list and have been approved by the Food and Drug Administration (FDA) as well as the Environmental Protection Agency (EPA) in the USA for food and beverage consumption [64]. The antibacterial properties of EOs make them a useful component of commercially preservatives. For example, some essential oils such as oregano or basil can be used as natural antifungals for the production of dry sausages without having detrimental effects on sensory properties [65,66]. In recent years, there has also been increasing interest in EOs as a component of antimicrobial packaging [67,68]. However, incorporation of EOs to food systems may be difficult due to the nature of these compounds. It should be noted that the results obtained relate to the effect of essential oils on DON in in vitro studies, while the situation may be different when the oils affect the toxin present in the composite matrix of raw or processed food. At this stage, without further detailed research, we are unable to determine the applicability of the tested essential oils in food. The main limitation to EO applications in food is the possible induction of an undesirable odor or flavor, the phytotoxicity risks, and the technological problems associated with their application [69].

## 5. Conclusions

Based on the performed tests, it can be concluded that the most optimal conditions for the reduction of deoxynivalenol content by the tested essential oils were as follows: 24 h, in the pH range of 3–6, and at 20 °C. The obtained results can provide the basis for further research on, among other things, explaining the mechanism of mycotoxin level reduction by essential oils, the durability of this process, and the resulting decomposition/binding products. This would allow for the use of safe, ecological, vegetable EOs to improve the quality and safety of food, especially cereal products.
